# Targeting electroencephalography for alcohol dependence: A narrative review

**DOI:** 10.1111/cns.14138

**Published:** 2023-03-08

**Authors:** Huiwen Zhang, Jiahui Yao, Cheng Xu, Chengyu Wang

**Affiliations:** ^1^ Department of Anaesthesiology General Hospital of Ningxia Medical University Yinchuan China; ^2^ Department of Anaesthesiology Shanghai Jiaotong University Affiliated Sixth People's Hospital Shanghai China

**Keywords:** alcohol dependence, event‐related oscillations (ERO), event‐related potentials (ERP), polysomnography (PSG), resting electroencephalography (REEG)

## Abstract

**Background:**

Electroencephalography (EEG)‐based electrophysiological techniques have made progress in diagnosing and treating alcohol dependence in recent years.

**Aims:**

The article reviews the latest literature in this field.

**Materials and methods:**

Alcohol dependence, which is common and prone to relapsing, poses a serious threat to individuals, families, and society. At present, the objective detection methods for alcohol dependence in clinic are not enough. As electrophysiological techniques developed in psychiatry, some researches on EEG‐based monitoring methods are of great significance in the diagnosis and treatment of alcohol dependence.

**Discussion:**

As electrophysiological techniques developed in psychiatry, some researches on EEG‐based monitoring methods such as resting electroencephalography (REEG), event‐related potentials (ERP), event‐related oscillations (ERO), and polysomnography (PSG), was reported.

**Conclusion:**

In this paper, the status of electrophysiological researches on EEG in alcoholics are reviewed in detail.

## Graphical abstract

EEG for alcohol dependence.

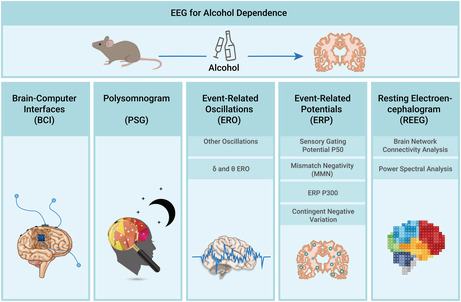

## 1 INTRODUCTION

Alcohol is a highly addictive substance, which is consumed by a large number of people. Alcohol consumption is now the third most common disease risk factor, after High blood pressure and smoking.^1^ According to the World Health Organization, approximately 3 million people died in alcohol misuse worldwide in 2016. Alcohol misuse poses significant challenges to public health in the United States, contributing to more than 95,000 deaths each year. Alcohol misuse also imposes a massive economic burden, with an estimated annual cost of $249 billion.^2^ Furthermore, nearly 15 million people in the United States are diagnosed with alcohol use disorder (AUD), a medical condition characterized by an impaired ability to stop or control alcohol use despite adverse social, occupational, or health consequences. Therefore, alcohol misuse is a major global public health problem.

The core symptoms of alcohol dependence are increased tolerance and withdrawal reaction, and the clinical treatment is mainly to relieve the symptoms. However, most patients still have strong drinking desire during abstinence even if they are combined with psychological intervention and isolation of environmental interference on the basis of drug treatment, so it is extremely difficult to maintain long‐term abstinence and easy to relapse. So far, relevant studies have not fully elucidated the mechanism of alcohol addiction and its relapse. Most studies suggest it may have something to do with the “reward system”, as well as the craving for alcohol. In clinical practice, subjective indexes such as scales are often used to quantify alcohol craving. Lack of objective evaluation indexes makes it impossible to accurately understand the degree of patients' craving for targeted treatment. Therefore, most patients drink again after discharge, which affects the therapeutic effect. Therefore, the objective evaluation method of alcohol dependence is helpful to improve the theoretical framework of alcohol addiction and guide the diagnosis and treatment.

In recent years, imaging, psychology, electrophysiology, and other fields have been exploring the related mechanisms of alcohol addiction. Electrophysiological technology has become a hot topic in alcohol dependence. Electroencephalography (EEG)‐based electrophysiological techniques have made progress for diagnosing and treating alcohol dependence in recent years. Therefore, the article reviews the latest literature in this field.

**FIGURE 1:**
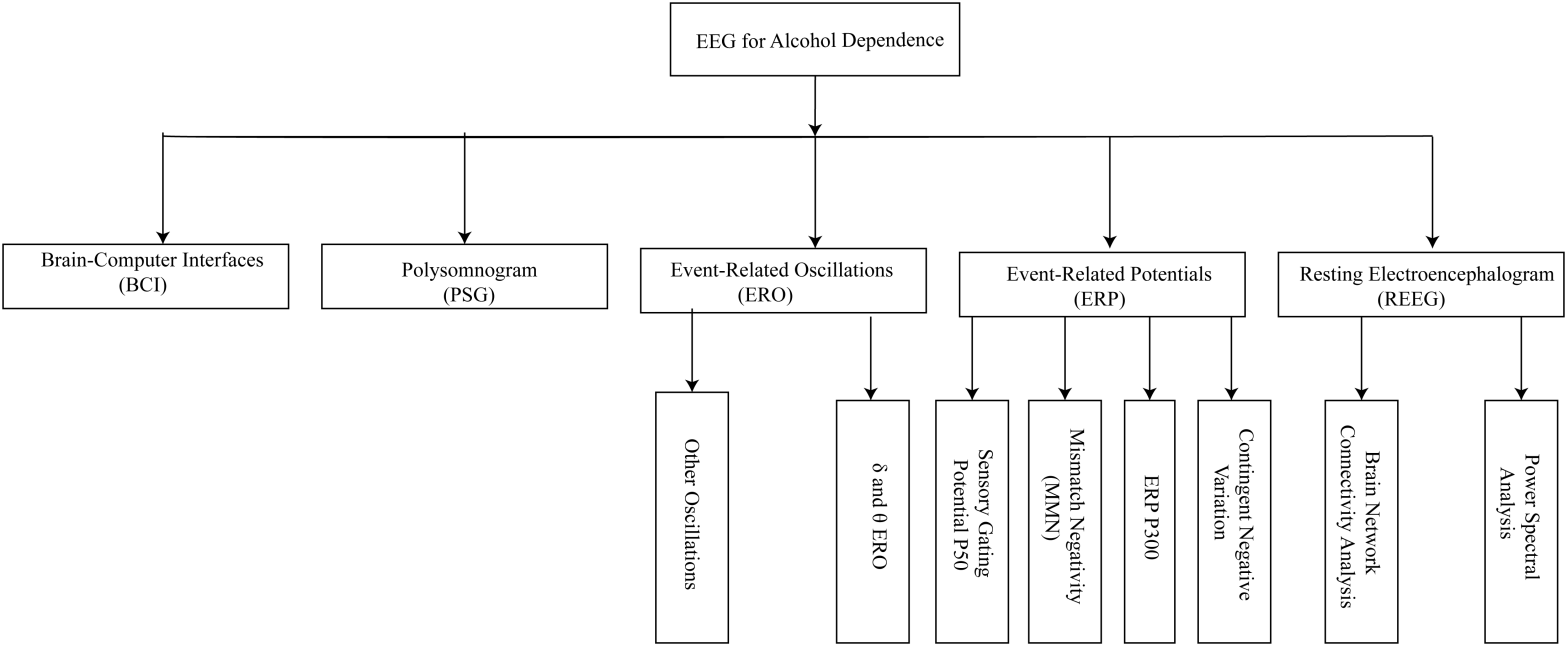
Technology roadmap.

Electroencephalogram (EEG) is a test that detects electrical activity in the brain using electrodes attached to the surface of the scalp. EEG signals provide a noninvasive and sensitive indicator of brain function during ongoing mental processes or task execution. EEG has the following advantages: (1) High temporal resolution: real‐time and ultra‐fast neurodynamics and information processing can be examined in the millisecond range; (2) Effectiveness: it acts as a direct neural activity; (3) Test–retest reliability: Excellent retest reliability within subjects and across laboratories; (4) easy to use and carry; (5) Lower cost recording system; (6) Suitable for a wide range of neurocognitive functions and abnormalities. EEG is used in almost all neurocognitive fields and diseases, including alcohol dependence.^3^, ^4^ In this paper, the brain electrical activity will be analyzed from the following four aspects: (1) resting electroencephalogram (REEG); (2) event‐related potentials (ERP); (3) event‐related oscillations (ERO); (4) polysomnogram (PSG; Figure 1).

## 2 RESTING ELECTROENCEPHALOGRAM (REEG)

Resting electroencephalogram (REEG) is an internal, task‐free, oscillating type of brain electrical activity, and usually quantified by frequency, amplitude, spectrum, or phase, which reflects to some extent the nature, intensity, and coherence of nerve impulses in different functional states of the brain.^5^ Spontaneous brain activity under the resting‐state, eyes‐closed condition has been increasingly identified as the brain activity correlate of cognition and behavior.^6^ REEG data is synthetic and can be divided into Alpha (*α*): 8–12 Hz, Beta (*β*): 12–30 Hz, Delta (*δ*): 0–4 Hz, Theta (*θ*): 4–8 Hz, Gamma (*γ*): 30–45 Hz. Different waves carry different kinds of physiological information and have been linked to brain activity. Current studies have found that α rhythm represents normal brain function, rhythm β is considered excitatory, while slow bands including δ and θ may be interpreted as inhibition.^7^ There is a speculation that γ rhythm is regulated by the θ, and it also indicates that the behavior of inhibitory interneurons is through the mechanism that γ rhythm is nested in θ rhythm.^8^ Therefore, analysis of resting‐state EEGs should help us to understand the basic brain function of patients with alcohol dependence.

### 2.1 Power spectral analysis (PSA)

Spectral energy analysis can quantify the energy value contained in different rhythms and is the analysis of the energy contained in a specific EEG rhythm. EEG energy reflects brain activity that records the location of electrodes. The difference in PSA between alcohol‐dependent patients and normal controls may reflect the pathological characteristics of alcohol‐dependent patients.

Compared with healthy people, alcohol‐dependent patients have a higher θ energy,^9^, ^10^, ^11^ and this unusual increase may reflect a reduced or obstructed ability to encode new information.^12^ Similarly, Rangaswamy et al. found an increase in θ energy at all scalp sites, prominent in the central, and parietal lobes of men and the parietal lobes of women, by comparing under closed eye conditions.^11^ Significant changes in θ are thought to be associated with cortical atrophy.^7^, ^13^

Rhythm α is the main REEG rhythm observed during the state of relaxation. Alcoholics showed a decrease in α‐energy, particularly in the occipital region, which indicated a lack of access to information from memory and attention.^7^

Among alcohol‐dependent patients, β energy has been studied more than γ energy. In alcohol‐dependent patients, whole‐brain β‐energy is elevated, whose increasement are independent of age and other clinical variables (such as timing and alcohol consumption).^7^, ^13^ However, gender affects β‐energy, which means the increase is more pronounced in male alcoholics than in female alcoholics.^14^ Meanwhile, β‐energy was elevated in the offspring of high‐risk patients.^15^ Changes in β‐energy have been interpreted as electrophysiological indicators of excitation‐inhibition imbalance in the cortex.^14^

In terms of prognosis, for alcohol‐dependent patients who drank again after abstinence and maintained abstinence, the δ and θ energy of those who drank again after abstinence increased. In the follow‐up 6 months later, it was found that there was no difference in δ and θ energy between the abstinence individuals and the normal control group.^7^ In rhythm α, the α‐energy decreased in patients with patient who drank again, while patient who abstinence from alcohol had increased α‐energy.^7^, ^10^ In β rhythm, β‐energy of patients who drank again was significantly increased.^10^ However, these changes may be related to whether medication (especially benzodiazepine), psychiatric disorders (hallucinations), seizures, and a family history of alcoholism. These factors can change β‐energy and affect classification accuracy.^13^ It has been concluded that these changes are less specific and cannot be interpreted as markers of recovery or the effects of drugs and withdrawal.^16^

### 2.2 Brain network connectivity analysis

EEG‐based quantities/features (such as PSA) provide information about specific brain regions. However, the brain as a whole needs to be studied on the network structure. Current researches on brain network connectivity include structural network connectivity and functional network connectivity. Structural network describes the anatomical connectivity of the brain, while functional network connection, including functional connection and effective connection.^17^ In the study of EEG, functional network connections are usually used. Functional network connection analysis methods, including coherence, synchronization, and phase locking, can be used to explore functional brain network connections between different brain regions represented by multiple recording electrode nodes.

The first step in constructing a functional brain network is to analyze the correlation of activity between different brain regions. The calculation of coherence takes into account the relationship between brain bioelectrical activity in different brain regions and provides their coupling information, which revealed subtle aspects of the dynamics of the brain network.^18^ In alcohol‐dependent patients, coherence was increased in the high frequency(6–7 Hz) at the θ.^19^ The increase of coherence may be related to craving. The result is similar to a study of smokers. The study found that θ coherence for nicotine cues was increased in the frontal lobe and parietal occipital regions, and it predicted changes in craving.^20^

Currently, linear techniques are commonly used to measure the relationship between recorded signals, but EEG signals are nonlinear signals. Therefore, sophisticated methods are needed to study them.^3^ Nonlinear research methods are getting more and more attention. Synchronization is usually used to represent the relative relationship of two or more systems over time. The likelihood synchronization gives a simple standardized estimate of the interdependence between two or more simultaneously recorded time series signals. Compared with other algorithms, likelihood synchronization has more advantages in analyzing non‐stationary signals such as EEG. Using the likelihood synchronization algorithm, deBruin et al. showed that heavy drinkers showed increased θ synchronization in the closed state compared to light drinkers, and increased θ coherence was also found in alcohol‐dependent patients.^9^ This similar effect in alcohol‐dependent patients and heavy drinkers hints at the synchrony effects of drinking on brain activity.

In addition, phase‐locked time analysis is also widely used in brain network analysis. EEG signal has three dimensions: frequency, amplitude, and phase. Whether EEG signals collected by different recording electrode nodes have phase synchronization through phase locking. Therefore, the analysis of brain networks needs to be integrated. Integrating coherence, phase, and spectral energy may reveal more about changes in brain activity during alcohol dependence. Tcheslavski et al. found that electroencephalogram energy, coherence, and phase synchronization were significantly reduced in alcohol‐dependence patients compared to controls.^21^ The future development direction also tends to integrate. Data mining for features based on the new classification algorithm can not only automate the entire process of screening, treatment, and prediction, but also achieve better accuracy, sensitivity, and specificity.

## 3 EVENT‐RELATED POTENTIALS (ERP)

Event‐related potentials is a kind of “time‐locked” electrical change induced by events with psychological significance, which reflects the neural electrophysiological phenomenon in cognitive processing. ERP can objectively evaluate advanced mental activities. Compared with fMRI and other neuroimaging techniques, ERP has better time resolution and can reflect the dynamic balance between excitation and inhibition of brain neural network in milliseconds.^3^ Most ERP components studied in psychiatry are CNV, P300, MMN, and P50.

### 3.1 Contingent negative variation (CNV)

Contingent negative variation reflects the negative potential of human brain complex psychological activity, which is closely related to the preparation, expectation, attention, motivation, and other mental activities of the human brain, and closely related to the attention retention ability of the subjects. CNV is induced by a warning‐command combined stimulus sequence, and consists of an orientation reaction (early component) and an expectation wave (late component).^22^ There are few reports on alcohol dependence‐related CNV studies. Cristini et al. found that in the Go /Nogo experimental paradigm, the late composition of CNV in the alcohol‐dependent group was significantly different from that in the control group, and its amplitude was at a higher level in the relapse patients.^23^ Relevant research needs further exploration.

### 3.2 ERP P300

In the study of ERP, P300 is one of the most widely applied, systematic, and theoretically mature technical. The Oddball paradigm is usually used to induce P300. Oddball paradigm is to randomly present subjects with two different stimuli, namely target stimulus and non‐target stimulus, in which the frequency of target stimulus is less than that of non‐target stimulus (the probability of target stimulus is usually 20%, the probability of non‐target stimulus is usually 80%) and the subjects only respond to the target stimulus.^24^ The P300 amplitude represents the amount of brain resources that person handled a problem, while the latency period represents nerve conduction velocity.^25^ ERP P300 is the most objective indicator applied to the measurement of cognitive function, which is of great significance for the diagnosis of cognitive impairment in patients with alcohol dependence.

Long‐term use of alcohol will affect patients' quality of life and social function, so early detection of cognitive changes in alcohol‐dependent patients, such as memory, attention, executive function, etc., plays an important role in improving the prognosis of patients. In combination with intelligent screening test C‐2.0(CASIC‐2.0), the P300 amplitude was significantly reduced in the alcohol‐dependent patients compared with the control group.^26^ Numerous studies have produced similar results.^3^, ^26^, ^27^, ^28^ These results indicated that the attention of these patients to the target stimulus decreased and the degree of cerebral cortex arousal was low. Kreusch et al. found that the P300 amplitude of alcohol‐dependent patients with low alcohol consumption was higher than that of patients with high alcohol consumption.^29^ In addition, some researchers found that the onset age of patients with alcohol dependence was negatively correlated with P300 amplitude. These results indicate that the pharmacological effects of alcohol can damage the normal attention of individuals, especially the decrease of the ability to allocate attention resources in the brain, and the greater the alcohol consumption, the more serious the damage, and the brain function is more serious in the older.

Some studies have shown that the morphology of P300 is mainly determined by individual physiological characteristics, such as callosity or the dissection characteristics of cranial bone thickness.^30^, ^31^ P300 showed up to 60% heritability in morphology.^32^ However, first‐degree relatives of alcohol‐dependent patients are usually high‐risk alcoholics, and their risk of developing alcohol dependence is much higher than that of normal people. Abundant information supports that genetic factors play an important role in susceptibility to alcohol dependence.

However, the specific gene expression of this susceptibility is difficult to be found, so the method of identifying the susceptibility gene of alcoholism from the genetic phenotype is used by many scientists. A genetic phenotype of alcohol dependence may be associated with the event‐related brain potential (ERP) P300 waveform. Comparing with those of healthy controls, the P300 amplitude of family members of alcohol‐dependent patients was higher.^33^ These results indicated that P300 was heritable and correlated with several chromosome regions by quantitative trait locus analysis.^34^ In addition, studies have shown that adolescents with lower P300 amplitude are more likely to develop alcohol dependence.^35^ These studies provide important support for P300 as a phenotype of alcohol dependence. Additionally, In Petit et al. ‘s study, 39 alcohol‐dependent patients were followed up after abstaining treatment, and it was found that 19 patients resumed drinking within 3 months. The amplitude of P300 in patients who resumed drinking was lower than that in patients who insisted on abstaining.^36^ Therefore, P300 is of great significance for the prognosis evaluation of patients with alcohol dependence.

### 3.3 Mismatch negativity (MMN)

Mismatch negativity is that the negative wave induced by the deviated stimulus that is larger than the standard stimulus when the deviated stimulus is inserted with a small probability in a series of repeatedly presented standard stimulus sequences in the non‐attentional state. MMN is usually elicited in the auditory Oddball paradigm, which calculates the ERP of the deviated stimulus minus the standard stimulus and presents the negative phase difference wave in 150–250 ms.

At present, there are conflicting conclusions about MMN related to alcohol dependence. It has been reported that MMN increased significantly in alcohol‐dependent patients compared with healthy controls, while some studies showed no abnormality. Grau et al. found that when the memory test interval was 0.4 s, MMN was not significantly different between the patient group and the healthy control group, while MMN was not observed in the patient group when the memory test interval was 5.0 s, which suggests that auditory memory is impaired in alcohol‐dependent patients, while the mechanism for automatic detection of stimulus changes is still preserved.^37^ Analysis of scalp current density (SCD) of MMN found that the left frontal lobe and right anterior temporal/posterior temporal region of the patients had decreased SCD, which indicates that the amount of alcohol consumed was negatively correlated with SCD intensity in these brain regions, and the duration of abstinence was positively correlated with SCD intensity.^38^ MMN can assist the clinical evaluation of alcohol dependence by reflecting the degree of neurological impairment.

### 3.4 Sensory gating potential P50

P50 was detected by conditional‐test mode, in which pairs of short pure tones S1 and S2 were used to induce positive phase potentials of about 50 ms respectively. S2‐P50/S1‐P50 represented P50 inhibition, which reflected the sensory gating mechanism of the central nervous system. Sensory gating defects may cause various mental symptoms related to attention. Studies have confirmed that P50 inhibition is abnormal to varying degrees in patients with schizophrenia,^39^ depression,^40^ cannabis abuse^41^ and cocaine dependence.^42^ In terms of alcohol dependence, patients also have abnormal P50, and still show impaired sensory gating after abstaining. It suggests that alcoholism leads to persistent deficits in inhibition of preattentional auditory processing in the brain.^43^ Studies related to alcohol intake have found that moderate doses of alcohol significantly reduce P50 sensory gating.^44^ Abnormal P50 inhibition should be associated with psychotic symptoms in alcohol‐dependent patients, and it is worth exploring its correlation.

## 4 EVENT‐RELATED OSCILLATIONS (ERO)

Event‐related oscillations is recorded during cognitive activity. ERO is a time‐frequency measure of electrical activity in the brain associated with sensory and/or cognitive events over time.^45^ ERO, especially ERO of δ, θ, and γ rhythmic activity, is considered to be characteristic of cognitive dysfunction in alcohol‐dependent patients.

### 4.1 δ and θ ERO

Brain oscillations at low frequencies (especially δ and θ rhythms) are thought to mediate a variety of cognitive processes.^45^ Jones et al. showed that alcohol‐dependent patients showed significant deficits in the induced δ and total θ energies, which contributed most to the P300 waveform.^46^ According to these studies, theθ oscillation forms the early part of the N200 and P300, while the δ oscillation forms the main part of the P300.^16^

θ is modulated by the emotional titer of the stimulus content. Aftanas et al. measured θ‐synchronization and de‐synchronization in healthy subjects exposed to stimuli with different emotional content and found time‐locked synchronization θ in the anterior and posterior heads within 200–500 ms after the presentation of the stimulus.^47^ This band can be used as a neuropsychophysiological marker of emotional processing. As for the effect of alcohol on θ in the emotional task, alcohol‐dependent patients seem to have lower θ energy in the emotional assessment task, no matter in the early assessment or in the later integration process.^48^ This low θ response to mood may indicate that alcoholics have exhibited some of the characteristic anhedonia of alcohol‐dependent patients.^49^ On the cognitive control dimension, Kamarajan et al. investigated the ability of alcoholics to induce EEG during Go/NoGo tasks. Results showed that under NoGo conditions, alcoholics had lower θ and δ energy levels than normal controls.^50^ Pandey et al. found that alcoholics had lower θ, δ, and α energy during Go/NoGo tasks compared to normal controls.^51^ In summary, there are cognitive deficits in executive and inhibition of motor response. Kamarajan et al. suggested that oscillations in cognitive processes could be used as an endophenic marker for alcoholism.^51^

In terms of prognosis, some scholars divide abstinence into short‐term abstinence and long‐term abstinence. Short‐term Abstinent Alcoholic(STAA) is defined as being in abstinent state for 3–6 months, while Long‐term Abstinent Alcoholic(LTAA) is defined as being in Abstinent for 6–12 months.^52^ By studying the non‐phase locked ERO_npl_(induced or non‐phase locked power, ERO_npl_) of alcohol‐dependent patients during abstinence, it was found that both STAA and LTAA individuals showed greater θ ERS(event‐related synchronization, ERS) energy to the target stimulus, and STAA individuals were more significant.^52^ This θ ERS energy is thought to be involved in working memory and attentional processes.^53^ Thus, larger θ ERS in STAA and LTAA suggest that alcoholics must pay more attention and have greater working memory to perform target detection in the Oddball task. Compared with LTAA, θ ERS in STAA is more likely to indicate that the compensatory mechanisms of alcohol‐dependent patients can overcome working memory and attention deficits. However, as the difficulty of the task increases, the increase in induced θ ERS energy may reach a critical value, which may eventually lead to the breakdown of the compensation mechanism. Therefore, for tasks that require more cognitive resources to be invested, more research is needed on alcoholics' non‐phase‐lock‐induced θ ERS to investigate how task demands affect differences between alcoholics and normal subjects. Finally, non‐phase lock‐induced θ ERS may be a candidate indicator for the recovery of brain function in treatment of alcohol dependence.

### 4.2 Other oscillations

During auditory memory tasks, acute alcohol intake reduces the α ERS response that occurs early in auditory encoding and increases the ERD(event‐related desynchronization, ERD) response that occurs late in retrieval. This suggests that alcohol has a destructive effect on EEG oscillations in both the θ and lower α frequency ranges during cognitive processing.^54^ γ ERO is related to selective attention and feature binding.^55^ In alcohol‐dependent patients and their high‐risk offspring under visual stimulation, early stimulus processing (0–150 ms after stimulation) reduced frontal lobe γ ERO energy during target processing, which indicates inadequate selective attention.^56^, ^57^

## 5 POLYSOMNOGRAM (PSG)

Polysomnogram is an electrophysiological monitoring method that continuously and synchronously records bioelectrical changes and physiological activities during sleep. Alcohol‐related sleep EEG studies have found that alcohol consumption has a significant effect on sleep regulation. Taking a large amount of alcohol before going to bed can shorten sleep latency. When blood alcohol content is high, sleep structure can be changed in the early night, sleep quality will deteriorate with the decrease of alcohol concentration in the late night.^58^ PSG tracing was performed on patients with alcohol dependence, and it was found that the total sleep time, sleep efficiency, and sleep phase transition of patients was significantly reduced, the sleep latency was significantly prolonged, and the duration of non‐rapid eye movement sleep was significantly shortened, which suggests that the sleep structure of patients with alcohol dependence was quite disorder.^59^ Other studies have found that the higher a patient's alcohol cravings in the early stages of withdrawal, the worse sleep quality and continuity.^60^ PSG sleep monitoring is helpful to comprehensively understand the severity of alcohol dependence disease, and may also predict the risk of relapse, which is worthy of extensive clinical application.

## 6 BRAIN‐COMPUTER INTERFACES (BCI)

A brain‐computer interface (BCI) is a direct connection between the brain and external electronic devices, which is operated by signals generated during brain activity.^61^ BCIs have exhibited a plethora of possibilities for use in various domains, including communication, synchronous control, asynchronous control,^62^ and rehabilitation.^63^ The electroencephalogram (EEG) is a popular choice for constructing BCI systems due to its cost‐effectiveness, non‐invasive nature, and portability. Throughout numerous BCI studies, growing attention has been dedicated to analyzing EEG of motor imagery.^64^, ^65^, ^66^, ^67^ In the field of alcohol dependence, BCIs can be used to monitor the brain activity of patients and detect the craving of alcohol. BCI also can be used to detect and analyze EEG signals, which can provide an effective approach for the diagnosis, assessment, and treatment of alcohol‐related disorders. Meanwhile, EEG signals also can be used to analyze the cognitive state of alcohol‐dependent. BCI is helpful to comprehensively understand the severity of alcohol dependence disease, and may also predict the risk of relapse, which is worthy of extensive clinical application.

## 7 SUMMARY

In this article, a comprehensive overview of the contribution of EEG‐based techniques to alcohol‐dependent patients and its associated challenges have been discussed. The purpose is to emphasize that future research can be guided in this context. Existing EEG methods have received little attention because most EEG studies are inefficient and the results are not reliable enough to be accepted. In addition, the small sample sizes used by these methods do not generalize their findings. Finally, the experimental paradigm and EEG data analysis methods related to alcohol dependence are relatively simple, and the neurocognitive and psychological significance of exploration is not deep enough. In future research, we can integrate the above monitoring methods and develop systematic assessment tools while gradually optimizing the electrophysiological technology, so as to comprehensively understand the condition of alcohol‐dependent patients, especially to trace the source of the neuroelectrophysiological lesions of craving.

## AUTHORS' CONTRIBUTIONS

H Z and J Y drafted the manuscript; C W designed the research; C X search article.

## CONFLICT OF INTEREST STATEMENT

The authors declare no conflict of interest.

## DISCLOSURES

All authors have nothing to disclose.

## Data Availability

Data sharing not applicable to this article as no datasets were generated or analysed during the current study.
